# *Mycobacterium tuberculosis* causing tuberculous lymphadenitis in Maputo, Mozambique

**DOI:** 10.1186/s12866-015-0603-5

**Published:** 2015-11-21

**Authors:** Sofia Omar Viegas, Solomon Ghebremichael, Leguesse Massawo, Matos Alberto, Fabíola Couto Fernandes, Eliane Monteiro, David Couvin, José Maiane Matavele, Nalin Rastogi, Margarida Correia-Neves, Adelina Machado, Carla Carrilho, Ramona Groenheit, Gunilla Källenius, Tuija Koivula

**Affiliations:** National Institute of Health, Ministry of Health, Maputo, Mozambique; Faculty of Veterinary, Eduardo Mondlane University, Maputo, Mozambique; Department of Clinical Science and Education, Karolinska Institutet, Stockholm, Sweden; Department of Microbiology, Public Health Agency of Sweden, Solna, Sweden; Pathology Service, Maputo Central Hospital, Ministry of Health, Maputo, Mozambique; Department of Pathology, Faculty of Medicine, Eduardo Mondlane University, Maputo, Mozambique; WHO Supranational TB Reference Laboratory, Tuberculosis & Mycobacteria Unit, Institut Pasteur de la Guadeloupe, Abymes, Guadeloupe, France; Life and Health Sciences Research Institute (ICVS), School of Health Sciences, University of Minho, Braga, Portugal; ICVS/3B’s, PT Government Associate Laboratory, Braga/Guimarães, Portugal

## Abstract

**Background:**

The zoonosis bovine tuberculosis (TB) is known to be responsible for a considerable proportion of extrapulmonary TB. In Mozambique, bovine TB is a recognised problem in cattle, but little has been done to evaluate how *Mycobacterium bovis* has contributed to human TB. We here explore the public health risk for bovine TB in Maputo, by characterizing the isolates from tuberculous lymphadenitis (TBLN) cases, a common manifestation of bovine TB in humans, in the Pathology Service of Maputo Central Hospital, in Mozambique, during one year.

**Results:**

Among 110 patients suspected of having TBLN, 49 had a positive culture result. Of those, 48 (98 %) were positive for *Mycobacterium tuberculosis* complex and one for nontuberculous mycobacteria. Of the 45 isolates analysed by spoligotyping and Mycobacterial Interspersed Repetitive Unit – Variable Number Tandem Repeat (MIRU-VNTR), all were *M. tuberculosis*. No *M. bovis* was found.

Cervical TBLN, corresponding to 39 (86.7 %) cases, was the main cause of TBLN and 66.7 % of those where from HIV positive patients.

We found that TBLN in Maputo was caused by a variety of *M. tuberculosis* strains. The most prevalent lineage was the EAI (*n* = 19; 43.2 %). Particular common spoligotypes were SIT 48 (EAI1_SOM sublineage), SIT 42 (LAM 9), SIT 1 (Beijing) and SIT53 (T1), similar to findings among pulmonary cases.

**Conclusions:**

*M. tuberculosis* was the main etiological agent of TBLN in Maputo. *M. tuberculosis* genotypes were similar to the ones causing pulmonary TB, suggesting that in Maputo, cases of TBLN arise from the same source as pulmonary TB, rather than from an external zoonotic source. Further research is needed on other forms of extrapulmonary TB and in rural areas where there is high prevalence of bovine TB in cattle, to evaluate the risk of transmission of *M. bovis* from cattle to humans.

**Electronic supplementary material:**

The online version of this article (doi:10.1186/s12866-015-0603-5) contains supplementary material, which is available to authorized users.

## Background

Tuberculosis (TB) ranks as the second leading cause of death from a single infectious agent, after the human immunodeficiency virus (HIV) [1]. In 2013, an estimated 9 million people developed TB and 1.5 million died from the disease, from whom 360 000 were HIV positive [1]. The African continent accounts for one quarter of all TB cases in the world and also has the highest rates of cases and deaths relative to population [1].

Mozambique is one of the high burden TB and HIV countries with a prevalence of HIV infection in adults of 11.5 % [2] and an estimated TB prevalence of 559 per 100 000 population. Fifty six percent of the TB patients in Mozambique are estimated to be HIV positive [1]. Among all reported TB cases in 2013, 9.8 % were extrapulmonary [1].

TB is caused by bacteria of the *Mycobacterium tuberculosis* complex. The *Mycobacterium tuberculosis* and the *Mycobacterium bovis* are the primary agents of the disease in humans and cattle respectively.

The TB epidemic in Mozambique is caused by an extensive diversity of *M. tuberculosis* spoligotypes with predominance of LAM, EAI, T and Beijing lineages [3]. To our knowledge, no information regarding lineages involved in extrapulmonary TB in Mozambique is available.

Bovine TB, caused by *M. bovis,* is the main zoonotic disease caused by mycobacteria, affecting cattle, other domesticated animals and several free or captive wildlife species. In Mozambiquethe overall prevalence of BTB in cattle is 13.6 % [4], varying from 0.98 % in Massingir [5] to 39.6 % in the Govuro district [6]. Little is known about the impact of bovine TB as a human disease in low income countries, particularly in HIV positive patients, including in Mozambique.

In Africa, bovine TB accounts for an estimated median proportion of 2.8 % (range 0 %–37.7 %) of all reported human TB cases [7].

Bovine TB is spread to humans, typically by ingestion of unpasteurized milk or contaminated meat, causing extrapulmonary TB, but can also be transmitted by inhalation of aerosols causing pulmonary TB [8, 9]. Several studies have detected *M. bovis* in tuberculous lymphadenitis (TBLN) cases [10–15], being the most common among all extrapulmonary TB cases.

In the present study, we explored the public health risk for bovine TB in Maputo, capital of Mozambique, by characterizing the isolates from TBLN cases, during one year, in the Pathology Service of Maputo Central Hospital.

## Results

### Demographic characteristics

**Fig. 1:**
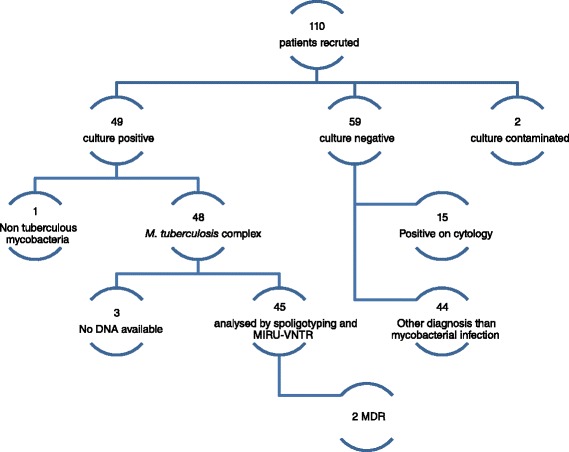
Chart showing patient’s recruitment, culture, identification and resistance results

Participants were recruited from July 2013 to July 2014 at the Pathology Service of Maputo Central Hospital, Mozambique. A total of 110 patients, suspected of having TBLN, were recruited to participate in the study (Fig. 1). From those patients, 45 isolates were analysed by genotyping methods, one isolate per patient. The demographic information of the 45 patients is summarized in Table 1. Among all, 28 (62.2 %) were male while 17 (37.8 %) were female.

**Table 1:** Demographic data of the 45 patients that were analysed by genotyping methods

Category	Total isolates	n (%)
Sex	Male	28 (62.2)
	Female	17 (37.8)
Age	18–45	39 (86.7)
	+45	6 (13.3)
HIV sero-status	Positive	30 (66.7)
	Negative	9 (20)
	Not tested for HIV	6 (13.3)
Previously TB treatment	Yes	15 (33.3)
	No	30 (66.7)
Contact with TB patients	Yes	15 (33.3)
	No	30 (66.7)

The patients’ median age was 33 years (11.1 SD) with a range of 18–75 years. Stratification according to age showed that 39 (86.7 %) of the patients were aged 18–45, while 6 (13.3 %) were above 46 years.

Fifteen (33.3 %) patients had been previously treated for TB and 15 (33.3 %) had had previous contact with TB patients.

### Site of sample collection

Cervical lymphadenitis was the main cause of TBLN in Maputo. Of the lymph node samples 39 (86.7 %) were collected from the cervical region, two (4.4 %) from axillary site and one (2.2 %) from inguinal region. Other sites were breast, chest and thigh (one case, 2.2 % from each site).

### HIV serology and drug resistance

Among the 45 patients, 30 (66.7 %) were HIV positive (19 males and 11 females), nine (20.0 %) were HIV negative and six (13.3 %) were not tested for HIV. Of the cervical TBLN cases, 26 (66.7 %) were HIV positive patients. Of the 61 patients with negative or contaminated culture results, 39 (63.9 %) were HIV positive, nine (14.8 %) were HIV negative and 13 (21.3 %) were not tested for HIV. No statistical association was found between HIV serology and cervical TBLN.

In Mozambique, being a mine worker is considered a risk factor for HIV [16], information related to previous work in South African mines was collected; among all positive cultures, four were from mine workers, of them two were HIV positive and two HIV negative.

**Fig. 2:**
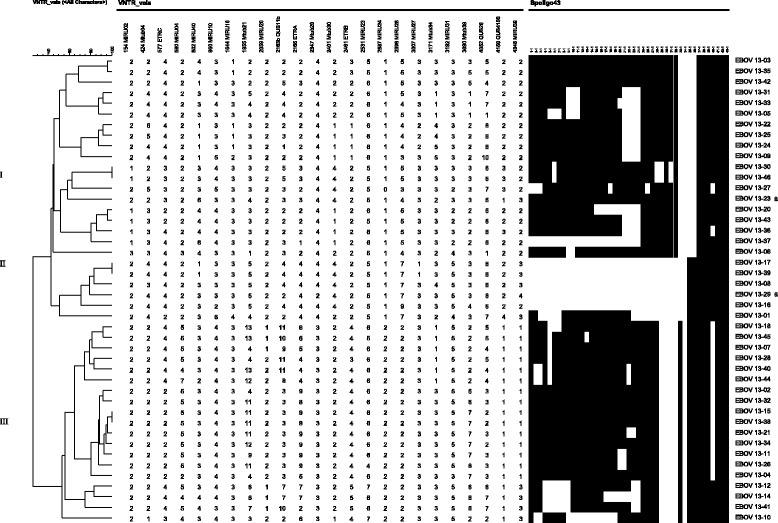
MIRU-VNTR and Spoligotyping dendrogram of the 44 *M. tuberculosis* strains from TBLN isolates from Maputo. The roman numbers indicates the MIRU-VNTR cluster in this study. a) Multidrug resistant isolates

Two patients, EBOV 13–23 (27 years, Lineage T1, SIT 53) and EBOV 13–29 (28 years, Lineage Beijing, SIT 1), both males, HIV positive and not previously treated for TB, were diagnosed with multidrug resistant (MDR) TB, defined as simultaneous resistance to isoniazid and rifampicin (Fig. 2).

### Culture and cytology results

Among all, 49 patients had a positive mycobacterial culture, giving a culture positivity rate of 44.5 %. Of them, 48 isolates were identified as *M. tuberculosis* complex and one as nontuberculous mycobacteria (NTM; 32 years, male, HIV positive). From the 59 culture negative patients there were an additional 15 (25.4 %) cases Ziehl Neelsen (ZN) positive on cytology (morphological evidence of mycobacterial infection). In the remaining 44 patients, based on cytology, there was a specific diagnosis other than mycobacterial infection.

Among the 48 culture confirmed TB cases, 45 isolates were analysed by spoligotyping and Mycobacterial Interspersed Repetitive Unit-Variable Number Tandem Repeat (MIRU-VNTR). For the remaining three isolates and for the NTM, DNA was not available, because there was no growth during the re-culture procedure (Fig. 1).

### Mixed infection isolate

**Fig. 3:**
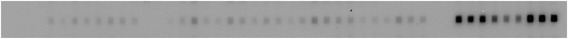
Spoligotyping pattern of the strain EBOV 13–19. The picture shows different intensities of the spacers

In patient EBOV 13–19 (male, 59 years, HIV positive), a spoligotype SIT 2117 (Manu 2; all spacers present except spacers 9, 10, 33 and 43) was observed (Additional file 1). This type of pattern may correspond to a mixed infection, due to concomitant Beijing and Euro-American lineage strains (the latter comprising H, LAM, X, and T lineages per spoligotyping defined clades). A mixed infection was defined as the occurrence of strains with different 24-loci MIRU-VNTR patterns at two or more loci in the same sample. We further investigated this isolate; by spoligotyping we could observe different intensities of the spacers in the spoligotyping pattern (Fig. 3) and by MIRU-VNTR we could observe double alleles in three different locus (MIRU26, Mtub21, and Mtub30), confirming a mixed infection pattern. Since it was confirmed as a mixed infection, the isolate EBOV 13–19 was excluded from further genotypic analysis.

### Spoligotyping

Spoligotyping is a simple, rapid and cost effective method for simultaneous detection and typing of the *M. tuberculosis* complex. It is the method of choice for strains with less than five copies of the insertion sequence IS*6110*, like *M. bovis* strains, which usually contain only one or two IS*6110* copies [17–19]. By spoligotyping, *M. tuberculosis* isolates are characterized by the absence of spacers 33–36, while *M. bovis* usually lack spacers 39–43 [20].

Spoligotyping was performed on 45 isolates. Of them, all were defined as *M. tuberculosis* and no *M. bovis* was found.

Among the 44 isolates (excluding the mixed infection isolate), 23 spoligopatterns were obtained. Three patterns corresponded to orphan strains that were unique in the SITVIT2 database, as opposed to 20 patterns from 41 patients that corresponded to shared-types (SITs), i.e. an identical pattern shared by two or more patients worldwide (within this study, or matching another strain in the SITVIT2 database), as shown in Table 2.

**Table 2:**
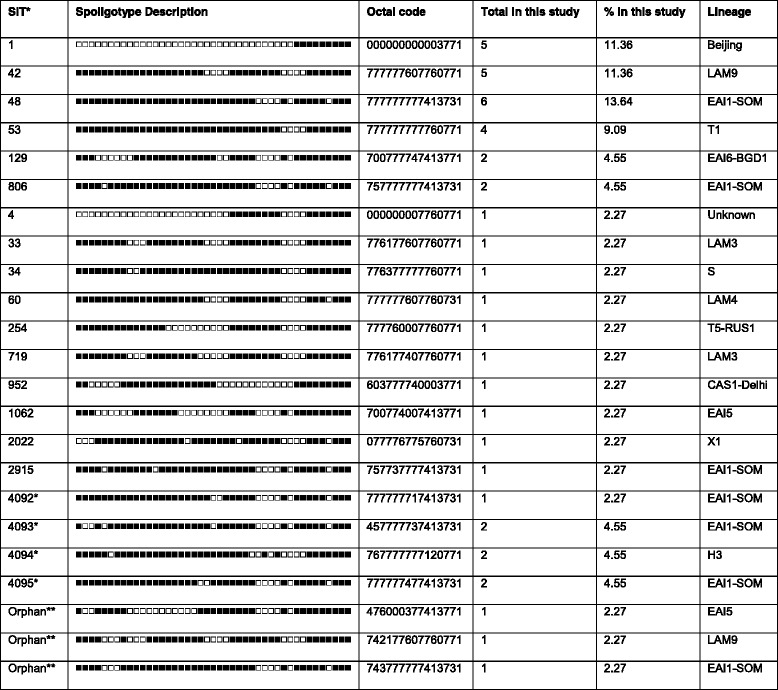
Shared Types and orphans description in the study, their percentage and lineage within the study

*Newly created SITs, either within the present study or after a match with an orphan in the database

**Orphans, unique in the SITVIT2 database

For each isolate, their binary/octal description, their lineages and SITs are summarized in Table 2. Four SITs (containing seven isolates) were newly created either within the present study or after a match with an orphan in the database. Nine patterns were in clusters, containing 30 isolates (2 to 6 isolates per cluster), amounting to an overall clustering rate of 68.2 % (30/44).

As shown in Table 2, the most common spoligotypes found in this study were SIT48 (East African-Indian_ Somalia; EAI1_SOM) with six isolates; SIT1 (Beijing lineage) and SIT42 (Latin America Mediterranean; LAM 9) with five isolates each and SIT53 (T1) with four isolates. The most common lineage was the EAI (*n* = 19; 43.2 %).

### MIRU-VNTR results

Figure 2 shows the MIRU-VNTR clusters and respective spoligotyping results obtained in this study. Among the 44 isolates analysed by MIRU-VNTR, a wide variety of patterns were observed. Only three clusters of two isolates each were formed, cluster I (Lineage H3, SIT 4094); cluster II (Beijing lineage, SIT 1); cluster III (Lineage EAI1_SOM, SIT 48). The remaining patterns were unique, i.e., did not cluster with any other isolate within this study.

### Phylogenetic analysis

Minimum Spanning Trees (MST) depicts evolutionary relationships between the *M. tuberculosis* genotypes in our study using spoligotyping and/or 24-loci MIRU-VNTR typing. The MST based on spoligotypes alone (Additional file 2A) showed isolated relatively well clustered into their respective lineages/sublineages. Isolates belonging to Beijing, EAI lineage and LAM were rather well organized in the three MSTs. However, on the MST based on 24-loci MIRU-VNTR alone (Additional file 2B), one may notice that the isolate represented by SIT254 or 24-MIT Or16 (lineage T5-RUS1), was better correlated with isolates belonging to LAM lineage. Furthermore, the isolate represented by SIT4 or 24-MIT Or30 (Unknown lineage) was also close to LAM lineage strains. Contrary to the MST based on spoligotypes alone (Additional file 2A) and on spoligotypes + 24-loci MIRU-VNTR (Additional file 2C), the MST based on 24-loci MIRU-VNTR alone (Additional file 2B) depicted a similarity between isolates belonging to Beijing lineage and the unique isolate (SIT952 or 24-MIT Or01) representing CAS1-Delhi lineage. For a better data mining of isolates related to 24-loci MIRU-VNTR information, one can refer to Additional file 1 showing the whole picture of association between the various genotypes.

## Discussion

In this study we presented for the first time the lineages of *M. tuberculosis* complex causing extrapulmonary TB in Maputo, Mozambique. Extrapulmonary TB is reported in 11.6 % of all TB cases in the country [1], the majority of them being TBLN.

Several studies have shown correlation between HIV infection and TBLN [21–23]. The synergies between TB and HIV infection [24, 25] have resulted in an increase in the incidence of TBLN and have further complicated TB control. In this study, the high prevalence of HIV positive patients (66.7 %) among TBLN cases, might suggest a rising trend of HIV infection associated with TBLN in Maputo. A possible explanation for the high rate of HIV among TBLN cases might be confounders associated with HIV. However, the numbers related to previous mine working did not allow the interpretation of potential associations with HIV and information related to alcohol, drug abuse, ex-imprisonment or smoking was not collected.

Laboratory detection of bovine TB is a challenge, particularly in low income countries. Microscopy for mycobacteria on the FNA is the initial diagnostic procedure for lymphadenitis in Mozambique; although it does not differentiate between *M. tuberculosis* and *M. bovis*, it is considered a reliable TBLN diagnostic method, including in HIV positive individuals [26–29]. Molecular typing methods for *M. tuberculosis* complex detection on FNA specimen are costly and require technical expertise, therefore, are not implemented as a routine method in the country, making the detection of bovine TB difficult.

In this study, among all TBLN suspects, 43.6 % were confirmed to have *M. tuberculosis* complex strains on culture and one was NTM, no *M. bovis* was found, showing that *M. tuberculosis* is the main cause of TBLN in Maputo. The additional 15 (25.4 %) cases that were positive on cytology might be due to infection by either *M. tuberculosis* complex or NTM which were not detected by culture.

These results are compatible with two studies conducted in the North of Ethiopia, where no *M. bovis* was detected and *M. tuberculosis* was identified as the main etiological agent in TBLN cases [30, 31]. On the other hand, another study conducted in Guji zone of Ethiopia, an area inhabited by pastoral and agro-pastoral communities whose livelihood is based on livestock production, among 173 isolates, three were *M. bovis*; the same study analysed 39 livestock samples, where one *M. tuberculosis* was isolated in camels, suggesting transmission between livestock and humans in this pastoral area. This last study emphasises the importance of an appropriate study area, where risk factors, including close contact between humans and livestock, and consumption of raw milk and meat by the communities, are present. This can be one of the reasons for the absence of *M. bovis* in the present study.

By spoligotyping, the main lineages of TBLN were the EAI, Beijing; LAM and T1; and the major SITs, were SIT48, SIT1, SIT42 and SIT53. These genotypes are also predominant in pulmonary cases in Mozambique [3], indicating that there are no differences in the population of strains in pulmonary and extrapulmonary cases. Similarities between pulmonary and extrapulmonary cases were observed in other countries, i.e. Ethiopia [31], Thailand [32], Madagascar [33] and Brasil [34].

In African countries, little is known about the common lineages responsible for extrapulmonary TB. A study conducted in the neighbouring South Africa, in children, showed similarities with our findings, stressing the proximity between the two countries. In the South African study, 21.2 % of the *M. tuberculosis* isolates belonged to the LAM lineage, and 20 % to the Beijing lineages [35]. In another study performed in Ethiopia, spoligotyping revealed that the most common spoligotypes in extrapulmonary TB were SIT54, SIT53, and SIT149 [36]; SIT54, Lineage T1 was also found to be common in TBLN cases from the present study.

The ancestral EAI lineage, most predominant in this study (42.2 %), and one of the prevalent lineages in pulmonary TB cases in Mozambique, with a predominance of 29.7 % [3]; is considered to be endemic in the Southern region of India [37, 38]. The migration link between India, particularly South India, and Mozambique arisen since the second half of the 19th century, when Indian traders practised the trade routes of the Indian Ocean, for transnational connections. The high prevalence of EAI lineages in Maputo might represent an indication of TB transmission between the two countries.

The Beijing lineage was also found to be one of the most common lineages in TBLN cases from this study. In South Africa, it was the second most common spoligotype found in extrapulmonary cases [35] and in Thailand, Beijing lineage was reported to be the most predominant in extrapulmonary TB cases, with 56 [39] and 57.9 % [40]. Further research is needed to evaluate whether Beijing lineage has any particular association with extrapulmonary TB.

We have also shown an association between the Beijing lineage and HIV infection [41], although in this study, perhaps because of the sample size, it was not possible to find any relationship between a particular lineage and HIV infection. Furthermore, analysis of the spoligotyping lineages did not show any association with a particular clinical expression of the disease (data not shown).

Based on MIRU-VNTR analysis, we could observe a wide diversity of patterns, within strain lineages, showing that TB lymphadenitis in Maputo is not caused by a particular strain but by a wide variety of strains, an indication that risk factors for developing TBLN are rather associated with host than *M. tuberculosis* strain.

In this study, a mixed *M. tuberculosis* complex infection was detected in one case of TBLN. Mixed *M. tuberculosis* infections, a potential obstacle for tuberculosis treatment and control, occurs when an individual is simultaneously infected with more than one strain of *M. tuberculosis* complex. In high TB prevalence settings, mixed infections are frequent, implying high reinfection rates and the absence of efficient protective immunity conferred by the initial infection [42].

In pulmonary TB cases from Mozambique, we have shown mixed infection with a Beijing and non-Beijing strain in two out of five Manu strains [3].

In South Africa, a study conducted in Cape Town, showed that 57 % of patients infected with a Beijing strain were also infected with a non-Beijing strain [42]. Other countries have also reported mixed infection within different strains from the *M. tuberculosis* complex, i.e. Botswana [43], China [44], Taiwan [45].

In this study, no *M. bovis* was found. In low income countries there are no effective animal TB control programmes and surveillance, and the epidemiological and public health aspects of infection due to bovine TB are scarce [7–9]. This situation is aggravated by the presence of additional risk factors such as human behaviour and the high prevalence of HIV infections [8, 9, 13]. In African countries, a median of 2.8 % (range 0 %–37.7 %) of all humans cases of TB are estimated to be caused by *M. bovis* [7]. Variances on prevalence are observed in different sites; those differences might be influenced by sampling, study area and diagnostic methods. Prevalence varies from; 17 and 4.4 % in Ethiopia [12, 46], 16 in Tanzania [47], 7 in Uganda [14], 3 in Ghana [48] and 15.38 % in Nigeria [49]. The genotyping findings from this study and the findings in pulmonary cases [3] indicate that the overall contribution of *M. bovis* to human TB in Maputo is minor. However, the present study has certain limitations. The small number of positive cases on culture might have reduced the chances of finding *M. bovis* as well as the statistical power and have affected the conclusions regarding the significance of the different variables and *M. tuberculosis* lineages. Furthermore, patients from this study are from urban or peri-urban areas of Maputo where livestock and consumption of unpasteurized milk is minor, thus, exposure to *M. bovis* is less.

The occurrence of zoonotic TB is greatly dependent on the presence of bovine TB in cattle. *M bovis* in cattle is very frequent in certain areas of Mozambique; a recent publication has demonstrated a high prevalence of bovine TB in cattle of 39.6 % (95 % CI 36.8–42.5) in one particular district of Mozambique [6]. Another study carried out in 2008 in the same region reported a bovine TB prevalence rate of 61.9 % (95 % CI: 55.8–67.8) [50]. Further research is needed on cases of abdominal TB and other forms of TB, and in pastoral areas, where the prevalence of bovine TB in cattle is known to be high in order to have a better answer about the public health importance of this zoonotic disease in Mozambique.

## Conclusions

*M. tuberculosis* was the main etiological agent of TBLN in Maputo. *M. tuberculosis* lineages in TBLN were similar to the ones previously reported to cause pulmonary TB, suggesting that in Maputo, cases of TBLN arise from the same source as pulmonary TB, rather than from an external zoonotic source.

## Methods

### Ethical considerations

Institutional permission to conduct the study was obtained from the National Bioethics Committee of the Ministry of Health in Maputo, Mozambique, reference number 216/CNBS/14. The patients were included after understanding the study and had signed an informed consent.

### Study setting

In Mozambique there are three pathology services, in Maputo, Beira and Nampula, each of them responding for the South, Central and North region of the country respectively.

The Pathology Service of Maputo Central Hospital is the only referral site in Maputo for diagnosing TBLN, patients suspected of mycobacterial infection were referred from different health units for diagnosis. During the study period, they have received 677 patients suspected of TBLN, of whom, 110 (16.2 %) were included in the study. The swellings observed were cervical, axillary or from other sites, either as a unilateral single or multiple mass or masses. Fistula formation could also been seen in certain cases.

Patients who also had pulmonary involvement were considered as extrapulmonary TB in our analysis.

### Patients and specimens

This study was conducted from July 2013 to July 2014 at the Pathology Service of Maputo Central Hospital in Maputo, Mozambique. A total of 110 patients with suspected TBLN and subjected to FNA were included in the study. For each patient, a questionnaire was applied.

Demographic and clinical information of the participants was collected by trained clinical nurses using a pre-tested questionnaire. HIV test results were collected from the medical records after obtaining written consent from the patients. Patients without HIV results were advised for testing and that was only performed after patients consent.

Only patients suspected of TBLN that consented and could give at least 0,1 ml of sample were recruited to participate in the study.. That was applied in order to have enough material for the routine smear performed in the unit, direct microscopy using conventional ZN staining and cytology, and subsequent assays to be performed within the study.

All material was collected by a Specialist Physician from the Pathology Service, using the FNA Cytology procedure in use in the Unit, as described below.

### Collection of lymph node aspirate and staining

Lymph node aspirate was collected as a routine procedure in the Pathology Service for all suspected of having TBLN. Briefly FNA was performed from a swollen superficial lymph node by using a sterile 23-gauge needle with an attached 10 ml syringe. The overlying area was cleaned with 70 % alcohol. Then the node was punctured by developing a negative pressure in the syringe. Multiple in and out passes were made by the needle without exiting the node. After removing the needle, in all cases, special ZN staining for acid fast bacilli was done and cytology was performed. The remaining aspirated sample was referred to The National TB Reference Laboratory for culture.

### Culture and identification methods

The cultures were performed using N-Acetyl-L-Cysteine-Sodium Hydroxide (NALC-4 % NaOH) decontamination method as previously described [51] and inoculated into three culture media: 1) Liquid culture (BD BBL™ MGIT™ Mycobacteria Growth Indicator Tube), 2) Löwenstein-Jensen slants (BD BBL™ Lowenstein-Jensen Medium) and 3) Stonebrinkslants (in house made-). All tubes were incubated at 37 °C..

All positive cultures were accordingly identified as *M. tuberculosis* complex or not using SD BIOLINE TB Ag MPT64 rapid test according to manufacturer’s instructions.

Isolates identified as *M. tuberculosis* complex were subjected to Line Probe Assay, GenoType MTBDRplus (Hain, Nehren, Germany), for detection of MDR TB as previously described [52].

### Spoligotyping

Standard spoligotyping [53] was performed generally as described by Kamerbeek and colleagues. Spoligotyping results were analysed and dendograms created using the BioNumerics Software ver. 7.5 (Applied Maths, Kortrijk, Belgium).

Spoligotyping patterns were also compared with the ones existing in the international Spoligotyping database SITVIT2, which is an updated version of SITVITWEB [54]. Shared international types (SITs) that were newly-created either within the present study or after a match with an orphan in the database, were assigned a new SIT number.

### Mycobacterial Interspersed Repetitive Unit – Variable Number Tandem Repeat (MIRU-VNTR) analysis

Standardized 24-loci MIRU-VNTR typing [55] was performed using the MIRU-VNTR typing kit (Genoscreen, Lille, France). The PCR-products were run with 1200 LIZ size standard (GeneScan, Applied Biosystems) on ABI3500 sequencers. Sizing of the PCR-fragments and assignments of MIRU-VNTR alleles were done with the GeneMapper software version 4.1 (Applied Biosystems) according to the manufacturers’ instructions. MIRU-VNTR were also compared with SITVIT2, and newly created 24-loci MIRU-VNTR International Types (24-MITs) were assigned.

### HIV testing

HIV testing was performed at the Sanitary Unit of enrolment according to the recommendations by the Ministry of Health, Mozambique. Two rapid HIV tests were used sequentially, Unigold Recombinant HIV (Trinity Biotech, Wicklow, Ireland) and Determine HIV-1/2 (Abbot, Tokyo, Japan). Samples were tested first with Determine and reported only when negative. Positive samples were confirmed with Unigold. All tests were performed and interpreted according to the manufacturer’s instructions.

### Statistical analysis

Statistical analysis was performed on Data Analysis and Statistical Software (STATA), version 13. Descriptive statistics was used for summarizing demographics data. Categorical variables were presented using frequencies and percentages. Bivariate analyses were performed for TB lymphadenitis versus HIV status, HIV status x lineage using chi-square test. Multinomial Logistic regression model were created with TB lymphadenitis as outcome and sex age and HIV status included as predictors. Interactions were tested and all were not statistically significant. Since no interactions were statistically significant, they are not presented.

### Phylogenetic analysis

Phylogenetic relationships were calculated using Multiple-Locus Variable-number tandem repeat Analysis (MLVA) Compare software version 1.03 (Genoscreen and Ridom Bioinformatics). MST were drawn from spoligotyping and 24-loci MIRU-VNTR typing, to better visualize probable relationships and dependencies between isolates. The phylogenetic trees connect each genotype based on degree of changes required to go from one allele to another (the distance numbers are visible on each edge). Solid black line denotes one unique change between two patterns, while solid grey line denotes 2 changes, bold dashed line denotes 3 changes, and thin dotted line represents 4 or more changes. The size of the circle is proportional to the total number of isolates. The colour of the circles indicates the phylogenetic lineage to which the specific pattern belongs. International Types (IT) numbers appear inside nodes. Both SIT and 24-MIT appeared inside nodes of the tree combining spoligotypes and 24-loci MIRU-VNTR.

## Additional files

Additional file 1:
**Full description of MIRU-VNTR and spoligotyping analysis in this study.** *Newly created 12, 15, or 24-MIRU International Types (MIT) were followed by an asterisk. Letters x, y, and z represent respectively MIRU-VNTR double alleles values of loci MIRU26, Mtub21, and Mtub30 (i.e. 7(+5), 5(+1), and 4(+2)). Letters A, B, C, and D, represent respectively MIRU-VNTR loci number of copies 10, 11, 12, and 13. **Mixed infection, Manu 2 isolate. (XLSX 15 kb) Additional file 2:
**MST illustrating evolutionary relationships between the** ***M. tuberculosis*** **spoligotypes of this study** **(** ***n*** **= 44 isolates).** Additional file 2A was drawn using spoligotyping alone, Additional file 2B was drawn using 24-loci MIRU-VNTR typing alone, and Additional file 2C was drawn using the combination of both spoligotyping and 24-loci MIRU-VNTR. (PPTX 402 kb)
