# Supplementary figures and images for: Mycobacterium tuberculosis causing tuberculous lymphadenitis in Maputo, Mozambique
Source: BMC Microbiol. 2015 Nov 21;15:268. doi: 10.1186/s12866-015-0603-5 (PMC4654834; doi:10.1186/s12866-015-0603-5)

## Slide 1
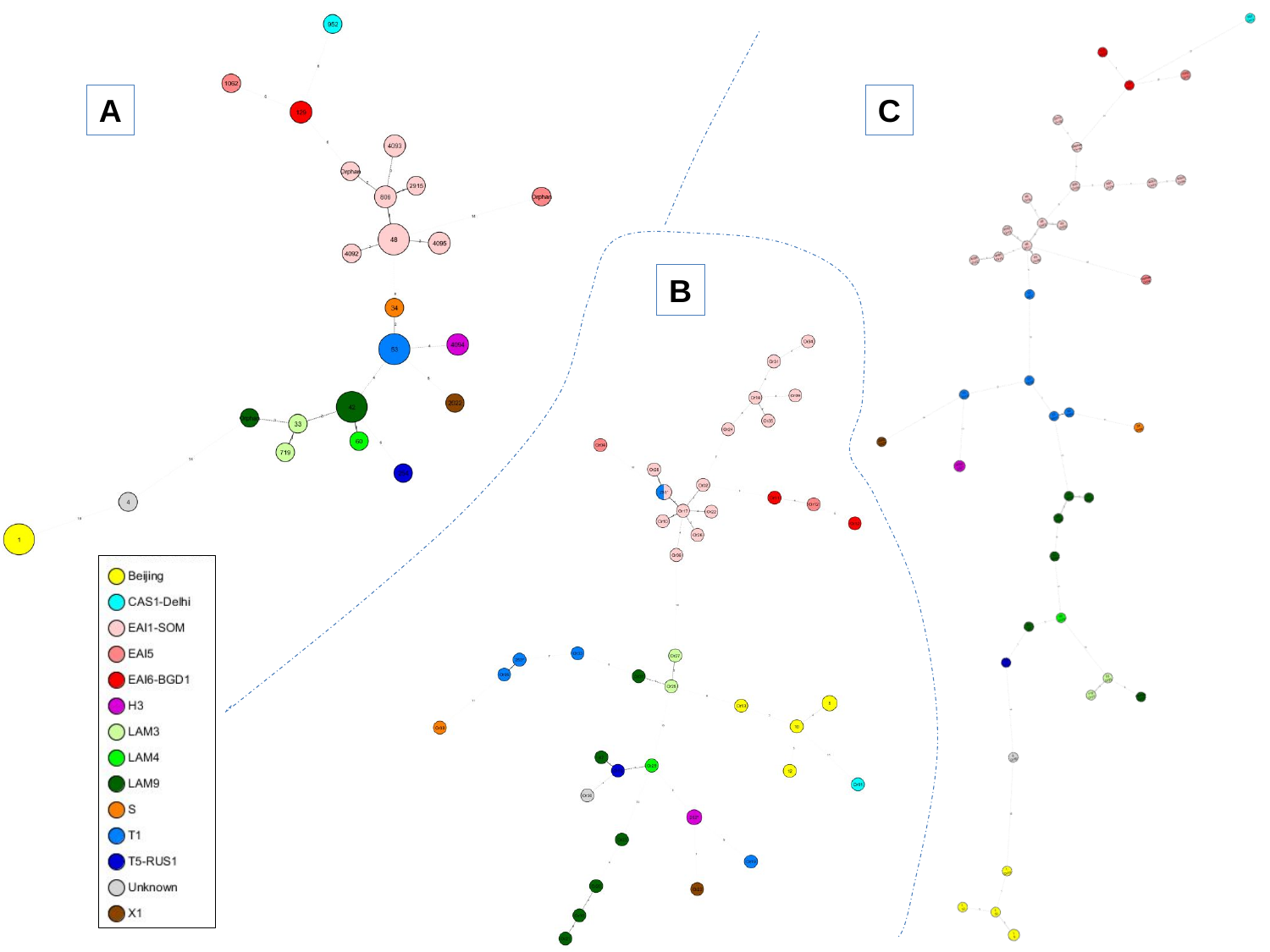

A
C
B

Supplement: Additional file 2: — MST illustrating evolutionary relationships between the M. tuberculosis spoligotypes of this study ( n = 44 isolates). Additional file 2A was drawn using spoligotyping alone, Additional file 2B was drawn using 24-loci MIRU-VNTR typing alone, and Additional file 2C was drawn using the combination of both spoligotyping and 24-loci MIRU-VNTR. (PPTX 402 kb) [file 12866_2015_603_MOESM2_ESM.pptx]
